# Optogenetic control of mitochondrial aggregation and function

**DOI:** 10.3389/fbioe.2024.1500343

**Published:** 2025-01-06

**Authors:** Luhao Zhang, Xuechun Liu, Min Zhu, Yuanfa Yao, Zhichao Liu, Xianming Zhang, Xin Deng, Yi Wang, Liting Duan, Xiaogang Guo, Junfen Fu, Yingke Xu

**Affiliations:** ^1^ Department of Endocrinology, Children’s Hospital of Zhejiang University School of Medicine, National Clinical Research Center for Children’s Health, Hangzhou, China; ^2^ Department of Biomedical Engineering, Key Laboratory of Biomedical Engineering of Ministry of Education, Zhejiang Provincial Key Laboratory of Cardio-Cerebral Vascular Detection Technology and Medicinal Effectiveness Appraisal, Zhejiang University, Hangzhou, China; ^3^ Innovation Center for Smart Medical Technologies and Devices, Binjiang Institute of Zhejiang University, Hangzhou, China; ^4^ Department of Cardiology, The First Affiliated Hospital, School of Medicine, Zhejiang University, Hangzhou, China; ^5^ Pharmaceutical Informatics Institute, College of Pharmaceutical Sciences, Zhejiang University, Hangzhou, China; ^6^ Department of Biomedical Engineering, The Chinese University of Hong Kong, Sha Tin, Hong Kong SAR, China

**Keywords:** optogenetics, mitochondria, aggregation, ATP, imaging

## Abstract

The balance of mitochondrial fission and fusion plays an important role in maintaining the stability of cellular homeostasis. Abnormal mitochondrial fission and fragmentation have been shown to be associated with oxidative stress, which causes a variety of human diseases from neurodegeneration disease to cancer. Therefore, the induction of mitochondrial aggregation and fusion may provide an alternative approach to alleviate these conditions. Here, an optogenetic-based mitochondrial aggregation system (Opto-MitoA) developed, which is based on the CRY2clust/CIBN light-sensitive module. Upon blue light illumination, CRY2clust relocates from the cytosol to mitochondria where it induces mitochondrial aggregation by CRY2clust homo-oligomerization and CRY2clust-CIBN hetero-dimerization. Our functional experiments demonstrate that Opto-MitoA-induced mitochondrial aggregation potently alleviates niclosamide-caused cell dysfunction in ATP production. This study establishes a novel optogenetic-based strategy to regulate mitochondrial dynamics in cells, which may provide a potential therapy for treating mitochondrial-related diseases.

## Introduction

Interactions among intracellular organelles are increasingly considered important regulators for many cellular processes, such as metabolite exchange (Helle et al., 2013), organelle remodeling (Guo et al., 2018), growth and organelle biogenesis (Friedman et al., 2011; Cohen et al., 2018). Among various subcellular organelles, the dynamic processes of mitochondrial fission and fusion, and its interactions with other organelles mediate many physiological processes, such as energy output (Seidlmayer et al., 2019), reactive oxygen species (ROS) production (Yu et al., 2006), lipid synthesis, calcium homeostasis (Cohen et al., 2018; Twig et al., 2008; Giacomello and Pellegrini, 2016), and cell death. For example, the bioenergetic roles of mitochondrial fusion have been identified. Knockout of mitofusins has been shown to impair ATP biosynthesis (Seidlmayer et al., 2019). Furthermore, the dysfunction of mitochondria and its interplay with other organelles such as endoplasmic reticulum (ER) has been demonstrated to be associated with neurodegenerative and metabolic disorders (Rieusset et al., 2016; Area-Gomez and Schon, 2016), cancer cell growth (Huang et al., 2023), and migration (Morciano et al., 2018).

Recently, the manipulation of organelle interactions and positioning has increasingly attracted the attention of researchers. Those techniques can be classified into chemical and genetic induction. Some chemicals can directly interact with protein complexes residing in organelle–organelle membrane contact sites (MCSs), influencing protein expression and regulating organelle interactions. For instance, LDC3/Dynarrestin, an aminothiazole derivative, has been demonstrated to enhance the interaction between mitochondria and ER via action on the tethering complex which consists of the ER protein vesicle-associated membrane protein-associated protein B (VAPB) and the mitochondrial protein tyrosine phosphatase-interacting protein 51 (PTPIP51) (Dietel et al., 2019). Chemically induced crosslinking of supramolecules artificially added onto the mitochondrial surface can also promote mitochondrial aggregation and fusion (Sun et al., 2020), while niclosamide has been shown to break intact mitochondria into fragments (Park et al., 2011). Genetic strategies take longer to function, regulating key proteins involving in organelle interaction such as mitofusins, dynamin-related protein1 (DRP1), VAPB, and PTPIP51 (Stoica et al., 2014) by gene silencing or overexpression.

In contrast to these strategies, optogenetics has been shown to be a promising approach in its rapid and reversible regulation of cellular processes with high spatial–temporal resolution. Optogenetic tools have been developed to control organelle interactions, positioning, and deformation in cells (van Bergeijk et al., 2015; Duan et al., 2015; Shi et al., 2018; Song et al., 2022). For example, the improved light-induced dimer (iLID) and stringent starvation protein B (SspB) system has been shown to tether the ER and mitochondria together (Tkatch et al., 2017). The co-expression of optogenetic elements, such as cryptochromes 2 (CRY2) and cryptochrome-interacting basic helix–loop–helix (CIB1), with specific targeting on organelle and molecular motors, have been used to control organelle trafficking and positioning (Berry and Wojtovich, 2020; Rensvold et al., 2022). Previous studies have used optogenetic modules to regulate the mitochondrial function by modulating iron pump activity, which ultimately controls mitochondrial membrane potential and perturbs ATP synthesis (Tkatch et al., 2017; Berry and Wojtovich, 2020). However, all these tools cannot control mitochondrial dynamics and make it hard to interpret the connection between mitochondrial morphology and function.

In this study, we present a novel optogenetic-based mitochondrial aggregation system (Opto-MitoA) that is based on the CRY2clust/CIBN light-sensitive module. We demonstrate that this optogenetic tool can rapidly control mitochondrial aggregation in cells and that its induced effects increase the energy-generating function of mitochondria and alleviate niclosamide-caused cell dysfunction. The Opto-MitoA may thus provide a new strategy for light-induced mitochondrial aggregation and fusion and for the potential treatment of mitochondrial-related diseases.

## Materials and methods

### Materials

Tom20-CIBN-GFP, Tom20-CIBN, and mCherry-CRY2clust plasmids were generated as per Duan et al. (2015). Niclosamide was purchased from Sigma Aldrich (St. Louis, MO, United States, No. N3510). 2′,7′-Dichlorofluorescin diacetate (DCFH-DA) for the assay of reactive oxygen species was purchased from Sigma Aldrich (St. Louis, MO, United States, No. D6883). CellTiter-Glo Luminescent Cell Viability Assay was purchased from Promega (Madison, WI, United States, No. G7571). Hoechst 33342 was purchased from Sigma Aldrich (St. Louis, MO, United States, No. 14533).

### Cell culture and transfection

The COS-7 cells were cultured in high-glucose Dulbecco′s modification Eagle′s medium (DMEM, Corning) with 10% fetal bovine serum (FBS, Gibco) and 1% penicillin G/streptomycin (Beyotime) at 37℃ in a humidified 5% CO_2_ incubator. The cells grown at 70%–90% confluency were transiently transfected with Lipofectamine 3000 (Thermo Fisher) according to the manufacturer′s instructions. After 18–24 h of transfection, the cells were used for imaging or functional evaluation experiments.

### Live cell imaging

Live cell imaging was performed as per Xu et al. (2016). COS-7 cells were cultured on 35-mm glass bottom dishes and imaged 18–24 h after transfection. The cells were imaged in pH 7.4 Krebs–Ringer bicarbonate HEPES imaging buffer (KRBH) containing 125 mM NaCl, 5 mM KCl, 1.3 mM CaCl_2_, 1.2 mM MgSO_4_, 20 mM D-Glucose, 25 mM HEPES, and 0.2% bovine serum albumin. Cells were kept in a thermostat-controlled chamber at 37℃ throughout the imaging process. An FV3000 confocal microscope (Olympus) with a 100 × NA 1.4 oil object was used for imaging. A 488-nm laser was used for visualizing GFP-tagged mitochondria and inducing CRY2clust oligomerization, and a 561-nm laser was employed to screen the oligomerization process of mCherry-labeled CRY2clust.

### Cellular ATP assay

The COS-7 cells were cultured in an opaque-walled 96-well plate overnight, with 100 μL cell culture per well. Both CellTiter-Glo Reagent and the plate were prepared and equilibrated to room temperature for 30 min. CellTiter-Glo Reagent was then added to the cell culture plate at 100 μL per well. An orbital shaker was used to mix the contents and induce cell lysis. The plate was incubated at room temperature for 10 min before record luminescence.

### Cellular ROS assay

The COS-7 cells were transplanted to a 96-well plate and cultured overnight. Cells were washed with PBS before incubation with 10 mM DCFH-DA for 30 min at 37℃. After rinsing with PBS three times, the plate was analyzed by a fluorescence microplate reader with excitation/emission at 485 nm/535 nm.

### Image analysis and statistics

Stacks of time-lapse images were processed and analyzed using ImageJ 1.51 s (National Institutes of Health). To quantify the degree of mitochondrial aggregation, the entropy of each image was normalized by the first frame of the time-lapse image sequences. All data were presented as the mean ± SEM and statistically tested by using Student’s t-test unless otherwise indicated. All analyzed experiments used biological replicates to compute statistical significance. In all statistical analyses, P < 0.05 was considered statistically significant.

## Results and discussion

### Rapid optogenetic control of mitochondrial aggregation in cells

**FIGURE 1:**
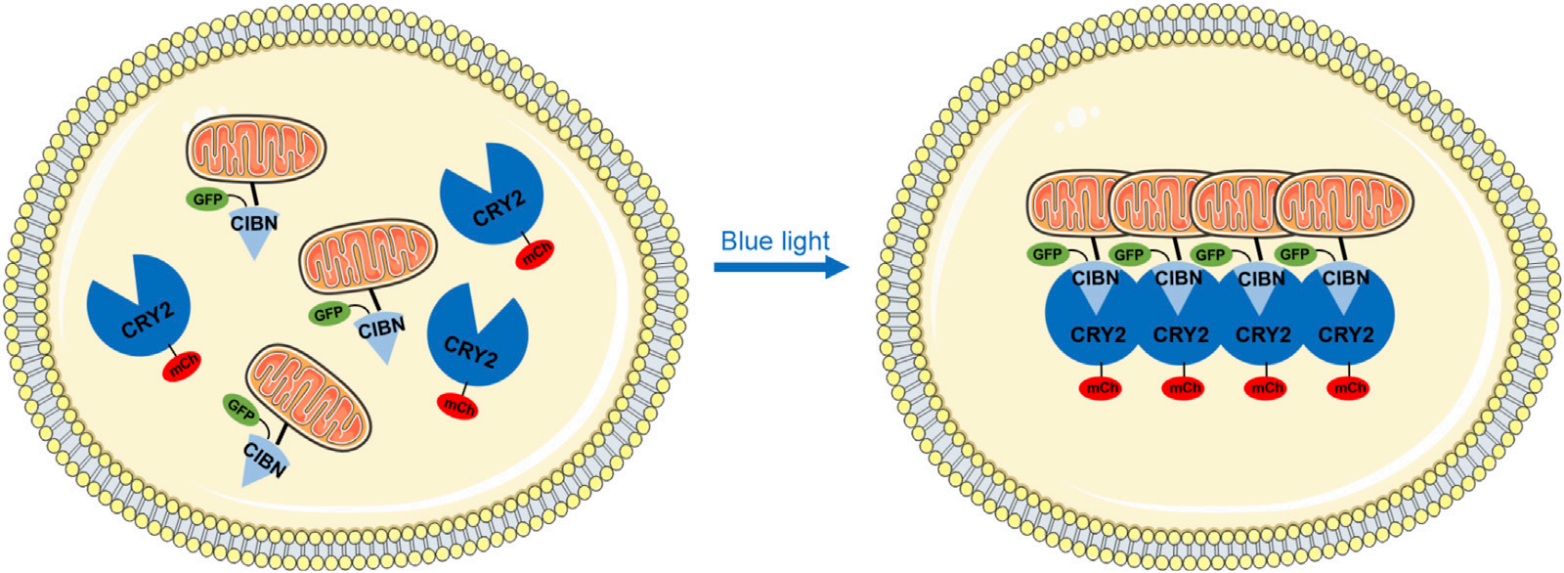
Schematic diagram depicting the optogenetic-based mitochondrial aggregation system (Opto-MitoA) in cells. In this schematic illustration, CIBN-GFP is specifically targeted at the mitochondria via the Tom20 domain. CRY2clust (CRY2) tagged with mCherry (mCh) is expressed in the cytosol. Upon blue-light illumination, CRY2clust formed protein clusters by homo-oligomerization, which further interacts with the CIBN to induce mitochondrial aggregation in the cell.

Mitochondria are highly dynamic cellular organelles that play an active role in calcium and damage-associated signaling, amino acids, and lipid metabolism and apoptosis (Rensvold et al., 2022; Araiso et al., 2022). The maintenance of mitochondrial integrity and homeostasis is extremely critical and is achieved through constant fusion and fission when exposed to metabolic and environmental stress (Adebayo et al., 2021). We proposed a novel system that employs blue light to rapidly control mitochondrial aggregation and to study how that perturbation affects cell function. This optogenetics-based mitochondrial aggregation system (Opto-MitoA) is based on blue-light-induced homo-oligomerization of CRY2clust (Park et al., 2017) and simultaneous hetero-dimerization between the CRY2clust and CIBN (Figure 1). CRY2clust is a C-terminal extension of CRY2PHR with nine-residue peptide, and CIBN is the N-terminal region of CIB1 (amino acids 1–170). Opto-MitoA consists of mCherry (mCh)-CRY2clust and Tom20-CIBN-GFP. We chose red fluorescent protein mCherry to track CRY2clust while not activating it. Tom20-CIBN-GFP is reported as targeting mitochondria and interacting with CRY2clust. Upon blue-light illumination, the CRY2clust forms protein clusters in the cytosol and interacts with the Tom20-CIBN-GFP to rapidly induce the aggregation of mitochondria in the cell (Figure 1).

**FIGURE 2:**
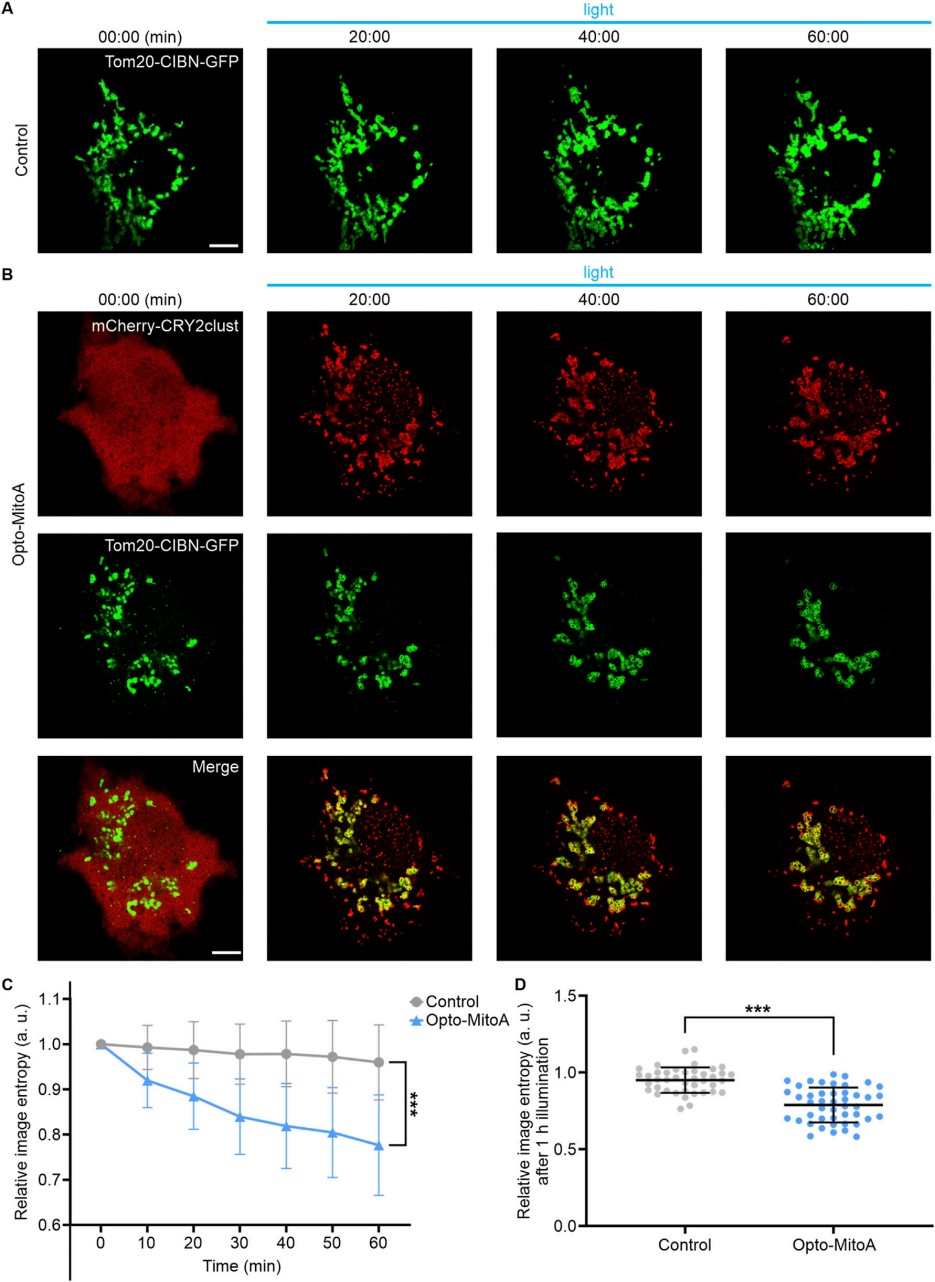
Rapid light-induced mitochondrial aggregation. The COS-7 cells were transfected either with Tom20-CIBN-GFP (Control group) or with both Tom20-CIBN-GFP and mCherry-CRY2clust (Opto-MitoA group). **(A)** Confocal images of COS-7 cells transfected with Tom20-CIBN-GFP before and after blue light illumination (representative time points shown). **(B)** Confocal images of COS-7 cells co-transfected with Tom20-CIBN-GFP and mCherry-CRY2clust before and after blue light illumination (representative time points shown). Scale bars: 10 μm. **(C)** Quantification of mitochondrial distribution at different time points by image entropy analysis. Data were statistically tested using one-way ANOVA. **(D)** Image entropy of mitochondrial distribution after 60 min of blue light illumination. Gray: control group. Blue: opto-MitoA group. n = 45 cells, *** indicates *P* < 0.001.

First, we confirmed the specific mitochondrial localization of Tom20-CIBN-GFP by performing co-localization experiments with MitoTracker Orange (Supplementary Figure S1). We then transfected COS-7 cells with only Tom20-CIBN-GFP plasmid or with both Tom20-CIBN-GFP and mCherry-CRY2clust plasmids to examine whether it induces mitochondrial aggregation. The cells were illuminated with a 488-nm laser (2 ms exposure time per pixel; 0.1 mW/cm^2^) at 10-s intervals to stimulate the oligomerization of CRY2clust and the dimerization of CRY2clust-CIBN, and the distribution and dynamics of mitochondria in cells were imaged by confocal microscopy. For cells only transfected with Tom20-CIBN-GFP, the GFP-labeled mitochondria rarely changed before and after 60 min of blue light illumination (Figure 2A, Supplementary Video S1). However, in the COS-7 cells co-transfected with Tom20-CIBN-GFP and mCherry-CRY2clust, we detected upon blue light illumination the robust formation of mCherry-CRY2 protein clusters in the cytosol and the concomitant formation of mitochondrial aggregation after 60 min of light activation (Figure 2B, Supplementary Video S2).

To quantify mitochondrial aggregation, we employed image entropy (Shannon, 1948) to measure the chaotic degree of mitochondrial distribution at different time points. The values of image entropy were calculated using a custom-written MATLAB program (Supplementary Figure S2). Image entropy is negatively correlated with the chaotic degree of mitochondrial distribution; thus, the induced mitochondrial aggregation would decrease the value of image entropy. Our results showed that the image entropy of the Opto-MitoA group had reduced nearly 20% compared with the control group after 60 min of blue light stimulation (Figures 2C,D). In summary, we demonstrated that our developed Opto-MitoA module is capable of promoting mitochondrial aggregation by using light.

### Opto-MitoA-induced mitochondrial aggregation is irreversible

**FIGURE 3:**
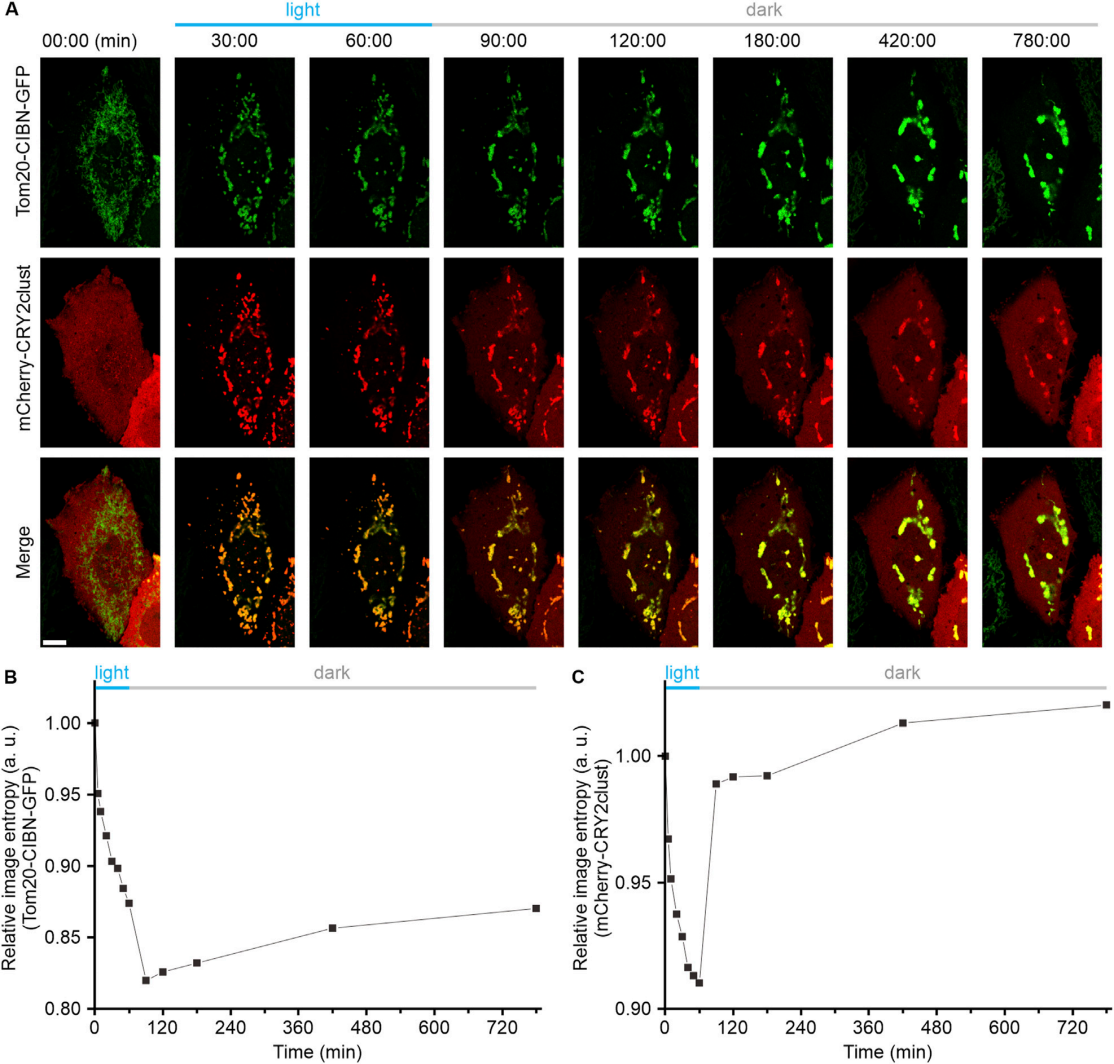
Light-induced mitochondrial aggregation is irreversible. COS-7 cells were transfected with both Tom20-CIBN-GFP and mCherry-CRY2clust (Opto-MitoA). The dynamics of mitochondria were imaged by confocal microscopy. **(A)** Representative confocal images showing the dynamics of mCherry-CRY2clust and the Tom20-CIBN-GFP-labeled mitochondria before and after blue light illumination, and recovery in dark conditions as time indicated. Scale bar: 10 μm. **(B)** Quantitative image entropy analysis of mitochondrial (GFP) aggregation before and after blue light illumination and recovery in dark conditions at different time points. **(C)** Quantitative image entropy analysis of CRY2clust (mCherry) aggregation before and after blue light illumination and recovery in dark conditions at different time points.

It has been well established that both light-induced homo-oligomerization of CRY2clust and hetero-dimerization between the CRY2 and CIBN are highly reversible (Park et al., 2017; Li et al., 2020; Xu et al., 2016). Hence, we performed experiments to determine whether Opto-MitoA-induced mitochondrial aggregation is reversible. The COS-7 cells were co-transfected with Tom20-CIBN-GFP and mCherry-CRY2clust plasmids, and the dynamics of mitochondria were imaged by confocal microscopy. As we previously demonstrated, blue light illumination rapidly induces the formation of mCherry-CRY2 protein clusters in the cytosol, and the dimerization of CRY2 and CIBN further promotes mitochondrial aggregation (Figure 3A). After 60 min of blue light exposure, we turned off the blue light. After another 30 min of recovery in the dark, we observed an increase of dispersed mCherry fluorescence in the cytosol, which indicates the reversibility of the CRY2clust and CRY2-CIBN modules. Images at 90 min show some red fluorescence still colocalized with green fluorescence (Figure 3A). We speculate that the aggregation of mitochondria increases the spatial hindrance of proteins, leading to a slower dissociation rate of the CRY2 and CIBN. Astonishingly, we found that the Opto-MitoA-induced mitochondrial aggregation was not reversible after recovery, although the expressed protein modules have been shown to be fully reversible. The mitochondria, as labeled with Tom20-CIBN-GFP, were still forming aggregates even after 720 min of recovery in the dark, and some proteins of mCherry-CRY2clust were trapped in aggregated mitochondria (Figure 3A). The image entropy analysis results demonstrated the progress of light-induced mitochondrial aggregation, clearly showing the irreversibility of Opto-MitoA-induced mitochondrial aggregation (Figure 3B), the reversibility of the homo-oligomerization of CRY2clust, and the hetero-dimerization between the CRY2 and CIBN (Figure 3C). The underlying mechanism of these interesting phenomena is not clear, and we suspect that the light-induced mitochondrial aggregation may directly promote mitochondrial fusion. Indeed, studies have shown that supramolecular crosslinking would induce mitochondrial aggregation and fusion, and the possibility of changes in mitofusion protein conformations would also affect mitochondrial fusion (Franco et al., 2016).

### Light-induced mitochondrial aggregation alleviates niclosamide-caused cell dysfunction

Mitochondrial dynamics and morphology are closely related to their functions. Chemically or genetically mediated structural perturbations of mitochondria would cause energy deficit, impair calcium ion homeostasis, and induce oxidative stress (Chinopoulos and Adam-Vizi, 2010). Niclosamide (5-chloro-N-(2-chloro-4-nitro-phenyl)-2-hydroxybenzamide) has been shown to modulate mitochondrial dynamics via the promotion of Drp1-dependent mitochondrial fragmentation (Park et al., 2011), which eventually increases cellular reactive oxygen species (ROS) content (Peng et al., 2023) and reduces ATP production (Sun et al., 2020; Zuo et al., 2024). Here, we sought to understand whether Opto-MitoA-induced mitochondrial aggregation could alleviate mitochondrial dysfunction caused by niclosamide treatment.

**FIGURE 4:**
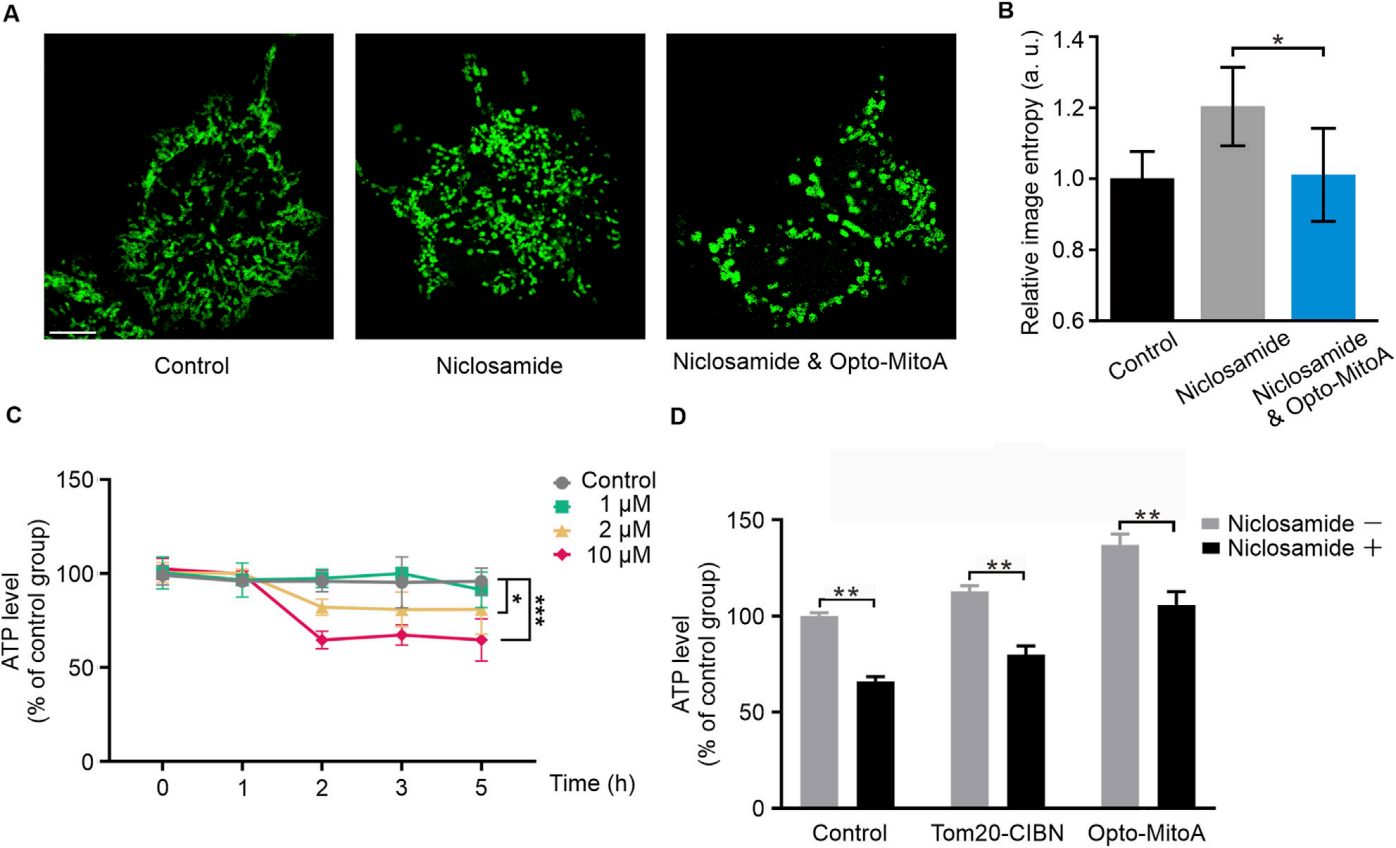
Opto-MitoA alleviates niclosamide-induced cell dysfunction. COS-7 cells were transfected either with Tom20-CIBN-GFP only or with both Tom20-CIBN-GFP and mCherry-CRY2clust (Opto-MitoA). The cells were treated with niclosamide or together with blue light activation, and the ATP production in cells was measured. **(A)** Confocal image samples of Tom20-CIBN-GFP-labeled mitochondria of control group, 10 μM niclosamide treatment group, rescue group with 10 μM niclosamide, and Opto-MitoA treatment. Scale bar: 10 μm. **(B)** Histogram of the image entropy of the control group, niclosamide group, and niclosamide and Opto-MitoA group. n = 5 cells. **(C)** ATP production in COS-7 cells treated with different concentrations of niclosamide and different incubation time. Data were statistically tested using one-way ANOVA. **(D)** Histogram of ATP production in control COS-7 cells with or without niclosamide treatment and in COS-7 cells expressing either Tom20-CIBN-GFP or with Opto-MitoA module, exposed with both niclosamide and light. Data are statistically tested using two-way ANOVA. *p*-value differences between each group are < 0.01. * indicates P < 0.05, ** indicates *P* < 0.01, *** indicates *P* < 0.001.

We found that of COS-7 cells with 10 μM niclosamide for 1 h caused mitochondrial fragmentation (Figure 4A). We then measured the ATP levels in these cells treated with different concentrations of niclosamide at different incubation times. Our results showed that niclosamide treatment caused ATP production to decrease in a concentration- and time-dependent manner (Figure 4D; Supplementary Figure S3). Treatment of COS-7 cells with 10 μM niclosamide for only 2 h reduced ATP production nearly 50% compared with untreated control cells (Figure 4D). Collectively, these results indicate that niclosamide treatment induces mitochondrial fragmentation and reduces ATP production in COS-7 cells.

We sought to investigate whether Opto-MitoA could improve mitochondrial functions and rescue mitochondrial dysfunctions induced by niclosamide. COS-7 cells were transfected with Tom20-CIBN-GFP and mCherry-CRY2clust plasmids. The cells were exposed with both niclosamide and blue light to induce the aggregation of fragmentated mitochondria in the cells. By using confocal microscopy, a higher density of mitochondria of both niclosamide and blue light groups was observed compared to the niclosamide-treated group (Figure 4A). Image entropy of both the niclosamide and blue light groups was significantly lower than the niclosamide-treated group (Figure 4B). These indicate the successful aggregation of fragmentated mitochondria. In the Opto-MitoA expressing cells, we found a higher basal concentration of ATP production compared with control cells and cells expressing Tom20-CIBN-GFP (Figure 4D). Although niclosamide treatment reduces ATP production in Opto-MitoA-expressing cells, its production is still at a level comparable to the control cells without drug treatment (Figure 4D). We further employed a fluorometric assay using dichlorofluorescein (DCF) to measure ROS levels in our samples. We found that cells treated with Opto-MitoA and niclosamide had a significant decrease in ROS production compared to cells treated with niclosamide after blue light illumination, indicating that the drug has the potential to rescue mitochondrial function (Supplementary Figure S4). Our results thus demonstrate that Opto-MitoA elevates the energy-generating function and decreases ROS production of mitochondria by promoting mitochondrial aggregation, which also functions to alleviate niclosamide-induced cell dysfunction in cells.

In summary, we developed a novel optogenetic-based mitochondrial aggregation system (Opto-MitoA) based on the CRY2clust/CIBN light-sensitive module. We validated its functionality of rapidly controlling mitochondrial aggregation in cells with its induced beneficial effects of increasing the energy-generating function of mitochondria and alleviating niclosamide-caused cell dysfunction. This tool seems to enable light-induced mitochondrial aggregation and fusion in cells and may have the potential for treating mitochondrial-related diseases.

## Data Availability

The original contributions presented in the study are included in the article/Supplementary Material; further inquiries can be directed to the corresponding author.
