# Supplementary material for: Optogenetic control of mitochondrial aggregation and function
Source: Front Bioeng Biotechnol. 2025 Jan 6;12:1500343. doi: 10.3389/fbioe.2024.1500343 (PMC11743975; doi:10.3389/fbioe.2024.1500343)
Supplement: Supplementary file 3 [file DataSheet1.docx]

**
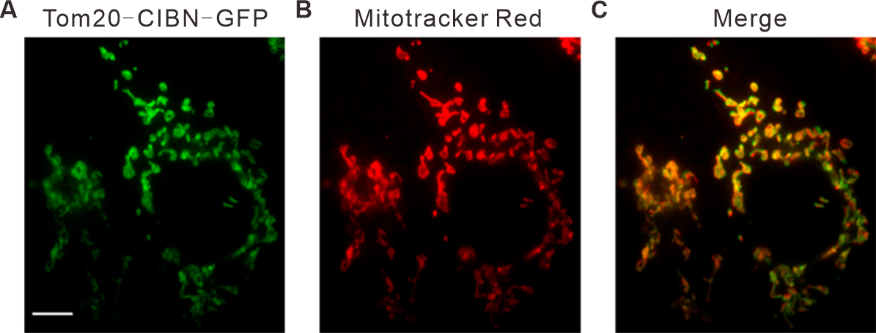
**

Figure S1. Colocalization of Tom20-CIBN-GFP and Mitotracker. A. COS-7 cells transfected with Tom20-CIBN-GFP (green fluorescent). B. COS-7 cells treated with Mitotracker Red (red fluorescent). C. Merged image of green fluorescent and red fluorescent. Scale bar: 10 μm.

**
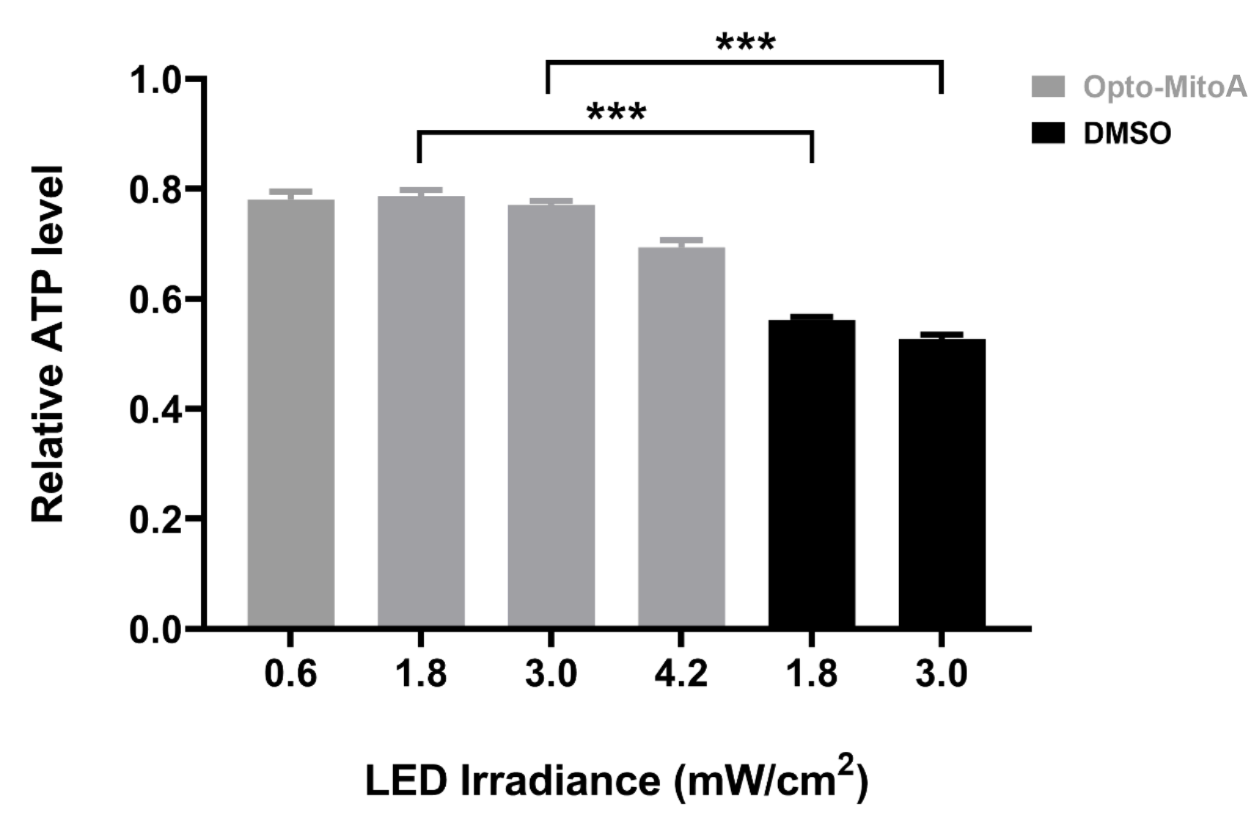
**

Figure S2. Effect of light intensity on ATP production of COS-7 cells. Opto-MitoA transfected and untransfected COS-7 cells were irradiated for 5 h with different intensity (0.6, 1.8, 3.0, 4.2 mW/cm^2^) of blue light. With the increase of light intensity, the ATP levels of cells in the same group did not change significantly, which indicated that the light intensity of 0.6 mW/cm^2^ is sufficient.


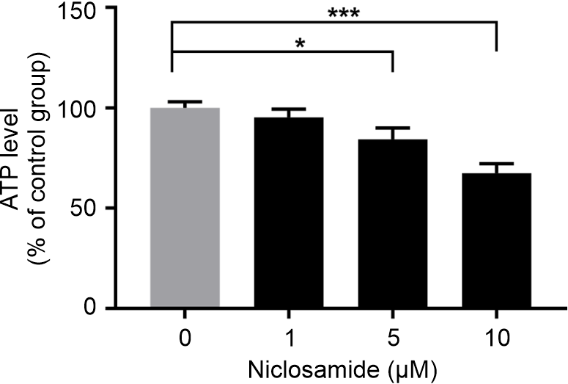


Figure S3. Histogram of the ATP production in control COS-7 cells incubated for 2 h with different concentrations of niclosamide.


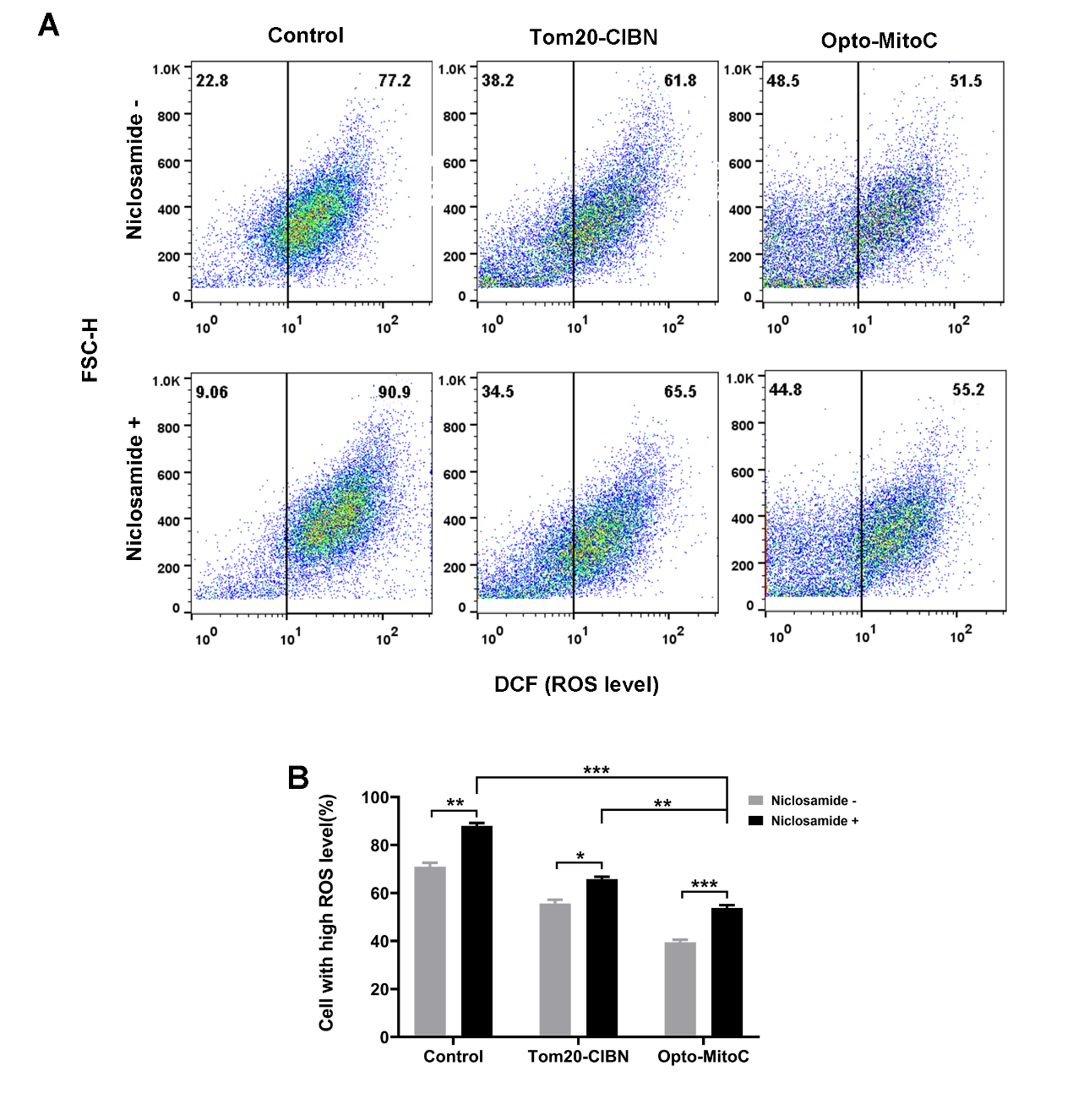


Figure S4. Regulation of ROS levels by optogenetics. A. Flow cytometry analysis of mitochondrial ROS levels measured by DCF after blue light illumination (0.6 mW/cm2, 5 h). Images from left to right were mock transfected COS-7 cells, cells transfected with Tom20-CIBN and Opto-MitoC group. Cells were treated with 10 μM niclosamide (Top) or not (bottom). B. Quantification of different groups of cells with high ROS level after treatment with niclosamide. *, P < 0.05; **, P < 0.01; ***, P < 0.001 (two-tailed t test).

**The code to calculate picture entropy**

clc

clear

close all

count=1;

saveh_x=[];

for num=1:205

filename=['C:\data’ , XXX , ’.tif’];

I=imread(filename);

[C,L]=size(I);

Img_size=C*L;

G=256;

H_x=0;

nk=zeros(G,1);

for i=1:C

for j=1:L

Img_level=I(i,j)+1;

nk(Img_level)=nk(Img_level)+1;

end

end

for k=1:G

Ps(k)=nk(k)/Img_size;

if Ps(k)~=0

H_x=-Ps(k)*log2(Ps(k))+H_x;

end

end

saveh_x(count)=H_x;

count=count+1

end
